# Artificial Intelligence-Driven Intrusion Detection in Software-Defined Wireless Sensor Networks: Towards Secure IoT-Enabled Healthcare Systems †

**DOI:** 10.3390/ijerph19095367

**Published:** 2022-04-28

**Authors:** Shimbi Masengo Wa Umba, Adnan M. Abu-Mahfouz, Daniel Ramotsoela

**Affiliations:** 1 Department of Electrical, Electronic and Computer Engineering, University of Pretoria, Pretoria 0002, South Africa; u17120056@tuks.co.za (S.M.W.U.); daniel.ramotsoela@up.ac.za (D.R.); 2 Council for Scientific and Industrial Research (CSIR), Pretoria 0184, South Africa

**Keywords:** Artificial Intelligence, deep learning, Internet of Things, intrusion detection, healthcare, security, Software-Defined Wireless Sensor Network, Wireless Sensor Network

## Abstract

Wireless Sensor Networks (WSNs) are increasingly deployed in Internet of Things (IoT) systems for applications such as smart transportation, telemedicine, smart health monitoring and fall detection systems for the elderly people. Given that huge amount of data, vital and critical information can be exchanged between the different parts of a WSN, good management and protection schemes are needed to ensure an efficient and secure operation of the WSN. To ensure an efficient management of WSNs, the Software-Defined Wireless Sensor Network (SDWSN) paradigm has been recently introduced in the literature. In the same vein, Intrusion Detection Systems, have been used in the literature to safeguard the security of SDWSN-based IoTs. In this paper, three popular Artificial Intelligence techniques (Decision Tree, Naïve Bayes, and Deep Artificial Neural Network) are trained to be deployed as anomaly detectors in IDSs. It is shown that an IDS using the Decision Tree-based anomaly detector yields the best performances metrics both in the binary classification and in the multinomial classification. Additionally, it was found that an IDS using the Naïve Bayes-based anomaly detector was only adapted for binary classification of intrusions in low memory capacity SDWSN-based IoT (e.g., wearable fitness tracker). Moreover, new state-of-the-art accuracy (binary classification) and F-scores (multinomial classification) were achieved by introducing an end-to-end feature engineering scheme aimed at obtaining 118 features from the 41 features of the Network Security Laboratory-Knowledge Discovery in Databases (NSL-KDD) dataset. The state-of-the-art accuracy was pushed to 0.999777 using the Decision Tree-based anomaly detector. Finally, it was found that the Deep Artificial Neural Network should be expected to become the next default anomaly detector in the light of its current performance metrics and the increasing abundance of training data.

## 1. Introduction

The ever-decreasing price of electronic devices coupled with the need to transfer automatically huge amount of data between remote locations has resulted in a paradigm known as the Internet of Things (IoT) [1]. The IoT is a system in which “things” (e.g., electronics and machines) communicate among them without the intervention of human beings to fulfill a specified task (e.g., controlling the temperature of an operating room). The different parts of an IoT system can be dispersed on a large field or placed in an environment (e.g., human stomach, hospital laundry room) where conditions such as acidity, humidity, and temperature do not allow the usage of wired communications [2,3]. To this end, Wireless Sensor Network (WSN) technologies are used in the applications of the IoT where wired communications are impossible to implement (e.g., global positioning system) or inadequate to use (e.g., wearable medical devices, ingestible sensors) [3,4,5,6,7,8]. Furthermore, the implementation of IoTs needs to take into account the number of sensors present in the network and the security threats such as the Denial of Service (DoS) attacks [9]. This fact underscores the need of establishing an adequate management of the network. To this end, the last decade has seen the development of a new paradigm referred to as the Software-Defined Network (SDN) [10,11]. The SDN model is drastically transforming traditional processes by providing a centralized control of the whole network making it easier to implement network-wide management protocols and applications such as data aggregation or cryptographic schemes [12,13,14,15,16]. The merging of the SDN model with the WSN model results in the Software-Defined Wireless Sensor Network (SDWSN) model.

**Figure 1:**
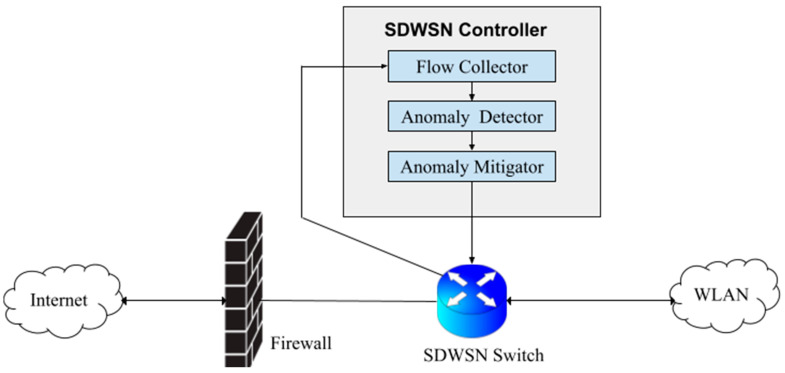
SDWSN-based IoTs IDS architecture (adapted from [29]).

Cryptographic schemes (i.e., symmetric, asymmetric cryptography and hybrid encryption) used in SDWSN-based IoTs are aimed at protecting them against security threats such as sybil attacks (i.e., an attacker steals the identity of legitimate sensor nodes) and unauthorized access [10,17,18,19,20,21]. Unfortunately, these schemes are not usually sufficient to ensure the integrity of communications in SDWSN-based IoTs [22,23,24,25,26]. To this end, the cryptographic schemes can be supplemented with an Intrusion Detection System (IDS) to monitor SDWSN-based IoT traffics and detect if an attack is being carried out by unauthorized entities [27,28,29]. The IDS is usually made up of three building blocks, namely, the flow collector, the anomaly detector, and the anomaly mitigator. Within the ambit of SDWSN-based IoTs, in order to optimize the network performance and monitoring, the IDS is programmatically deployed as a software on the controller. Figure 1 depicts the overall architecture of an IDS deployed on the SDWSN-based IoT controller.

The function of the flow collector in the IDS is to gather all flow features (e.g., source node name, number of failed login and connection time) and forward them to the anomaly detector [23,27,30]. The anomaly detector plays a central role in the IDS by using the features obtained from the flow collector to assign a class to the flow (e.g., sybil attack, normal traffic). The function of the anomaly mitigator is to take a stand (e.g., pass on or do not pass on the flow) given the class assigned to the flow by the anomaly detector [31]. The work in this paper will revolve around the anomaly detector given that this specific component constitutes the brain of the IDS because the decision to assign a class to a flow occurs in it. It is noteworthy that the terms “anomaly detector” and “classifier “are sometimes used interchangeably in the literature to simplify the text. In the same vein, the terms “SDWSN” and “SDWSN-based IoT” are used interchangeably in the literature.

Various approaches have been put forward in the literature as IDSs in SDWSNs [27,32,33,34,35,36]. Amid these approaches, the IDSs using as anomaly detector either a Decision Tree (DT), a Naïve Bayes (NB) classifier or an Artificial Neural Network (ANN) are widely used in the literature because they are relatively easier to implement while being very performant on classification tasks [32,33,34,35,36,37,38]. It is noteworthy to highlight that utterly disparate datasets were used in these published works to train the aforenamed anomaly detectors and for this reason, the performances achieved by an anomaly detector on one dataset could drastically dwindle on a different one. Furthermore, in the case of safety or mission critical networks (e.g., heart rate monitoring, automated insulin delivery) [39,40], on one hand the security constraints can prevent the network from using a cloud-based controller, whereas on the other hand the miniaturization constraints can limit the physical size and the memory capacity of the controller while the performance specifications can require a low latency. For these reasons, there is a need to choose judiciously an anomaly detector presenting the fastest execution time, the lowest memory size and energy consumption to guarantee the best trade-off between security and performance for safety or mission critical SDWSNs [31,41,42,43,44]. An additional remarkable observation is the fact that given that the SDWSN is a new paradigm, there is not a substantial body of literature related to the intrusion detection in SDWSNs. The Network Security Laboratory-Knowledge Discovery in Databases (NSL-KDD) dataset [45] is used in this paper to train an NB based anomaly detector, a DT based anomaly detector and a deep ANN based anomaly detector, respectively. It is noteworthy to point out that the state-of-the-art performance metrics established on the NSL-KDD dataset were obtained using a Least Square Support Vector Machine-based (LSSVM) IDS on which a Filter-based Mutual Information Feature Selection (FMIFS) scheme was implemented [36]. The LSSVM − IDS + FMIFS framework was able to yield the best accuracy (in binary classification) and best F-scores (in multinomial classification) when 18 features were selected. One of the goals of the present paper is to establish state-of-the-art performance metrics by using all 41 features found in the NSL-KDD dataset.

## 2. Aim of the Paper

This paper is an extended version of our works [28,29] published in the proceedings of the IEEE 28th International Symposium on Industrial Electronics (ISIE). As it was the case in our previous works, the accuracy, the F-score, the prediction time, the run time, and the memory size are used in this paper to make a fair comparison between these three anomaly detectors (i.e., DT, NB, and Deep ANN) and give orientations on the choice of the adequate approach to be used as the IDS for SDWSNs of different sizes. Our previous works were extended by introducing in this paper the multiclass classification besides the binary classification and given that the accuracy and the F-score are the performance metrics used to evaluate the state-of-the-art IDS in the body of literature [36], a particular focus was placed in this work on building algorithms that would yield state of the art accuracies (binary classification) and F-scores (multinomial classification). Furthermore, the underlying algorithms behind each of the three anomaly detectors were briefly described. Most importantly, example of applications in the healthcare sector were proposed and conclusions were drawn after evaluating, discussing, and benchmarking against the state-of-the-art approaches the performances of the three anomaly detectors developed in this paper.

## 3. Methods

### 3.1. Data and Performance Metrics

The NSL-KDD dataset is an upgraded and reduced version of the KDD cup 1999 dataset [46]. The NSL-KDD dataset was introduced to mitigate a number of issues (e.g., elimination of redundant records) found in the KDD cup 1999 dataset with the aim of speeding up and simplifying the development of anomaly detectors while bettering their performances. The accuracy, the precision, the recall, and the F-score [47,48] are four metrics widely used in the literature to gauge the performance of classifiers. By definition, the accuracy represents the ratio between the correctly classified records and all classified records. In the case of an anomaly detector performing a binary classification between attacks and normal traffics, the True Positive (TP) represents the proportion of attacks correctly classified; the True Negative (TN) represents the proportion of normal traffics correctly classified; the False Positive (FP) represents the proportion of normal traffics incorrectly classified and the False Negative (FN) represents the proportion of attacks incorrectly classified. For this reason, the accuracy can be mathematically formulated as a function of TP, TN, FP, and FN. From a mathematical point of view, the precision and the recall can also be formulated in one way or another as functions of TP, TN, FP and FN [48]. The F-score is a metric that combines the recall and the precision to gauge the performance of a classifier.

Table 1 gives the performance metrics commonly used in an anomaly detector and their respective formulas. It should be noted that the accuracy is the metric the most used for assessing the performance of anomaly detectors. Additionally, it should be noted that the values of the aforementioned performance metrics can be expressed as a percent or as a normalized number (a number between 0 and 1). In this paper, these performance metrics are expressed as normalized numbers. Furthermore, in order to assess the effectiveness of different anomaly detectors, besides the aforementioned performance metrics, we will use the time required to train and test an anomaly detector (i.e., run time), the time required to predict all records in the test set (i.e., prediction time) and the memory size of the anomaly detector. The times recorded on each anomaly detector, the performance metrics and the memory size of this latter will be compared to their equivalent in the two other anomaly detectors considered in this paper to give orientations on the choice of the adequate approach to be used as the anomaly detector of the IDS for a given SDWSN. It is noteworthy that the accuracies and the F-scores achieved by the anomaly detectors will be compared to the state-of-the-art accuracy and F-scores found in the literature (i.e., LSSVM − IDS + FMIFS framework) [36] in order to determine if these latter have been increased by an anomaly detector developed in the present paper. In the same vein, the accuracy will be used for the binary classification case while the F-score will be used for the multinomial classification case.

### 3.2. Analysis

The NSL-KDD dataset is made of records belonging to five classes, namely, normal traffics, Denial of Service (DoS) attacks, User to Root (U2R) attacks, Remote to Local (R2L) attacks and probing attacks [30,49,50,51,52,53]. Each record consists of 41 features and a label (one of the five aforenamed classes) that demarcate it from other records. Table 2 shows the distribution of these records. A DoS attack consists of crashing a network by flooding it with traffics so that the genuine requests will not be satisfied. A probing attack consists of inspecting a network in order to discover its weaknesses and exploit them to obtain unauthorized access. An R2L attack consists of an attacker intruding in the traffic of the network in order to obtain user (node) privileges. A U2R attack consists of a node trying to obtain superuser (i.e., controller in the contest of SDWSN) privileges in order to comprise the entire network. It is noteworthy that multiple attacks are launched concurrently in the case of probing and DoS attacks in opposition to the case of U2R and R2L attacks. For this reason, the U2R and R2L attacks are largely outnumbered by the DoS and probing attacks in Table 2.

### 3.3. Anomaly Detectors

#### 3.3.1. Decision Tree

**Figure 2:**
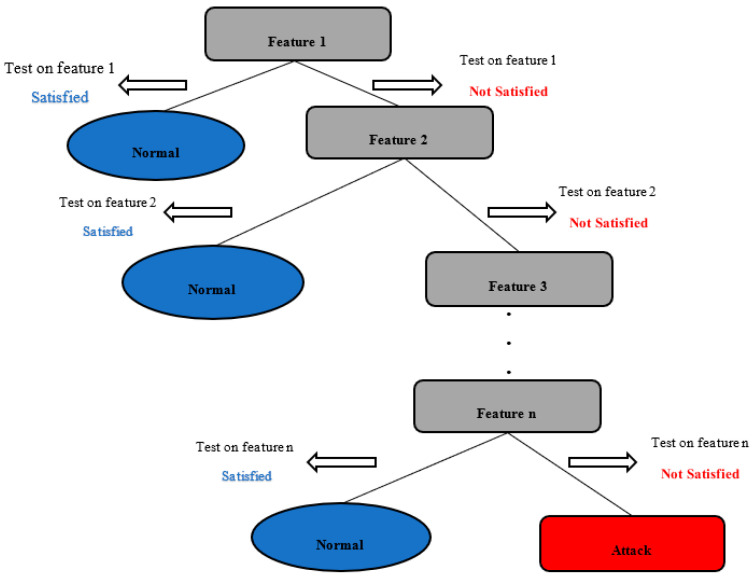
DT algorithm for binary classification.

The DT can be defined as a predictor *d*: U→Y [47]. The goal of the predictor *d* is to forecast the label *y* of a sample *u* by learning decision rules inferred from the training set while using a tree representation to this end. The tree representation starts by a root node and goes all the way to each leaf node. It is noteworthy that in the case of a classification problem, the best (i.e., most important) feature is always placed at the root node of the tree representation while each leaf node contains a specific label. A feature selection technique such as the information gain, the Gini index, the principal component analysis, or the genetic algorithm is usually used as the criterion to decide the importance of a feature in a given dataset [47,54,55]. Figure 2 shows the generic diagram of a DT algorithm in the case of a binary classification of traffic flows in SDWSN-based IoTs.

The DT algorithm allots the best attribute to the root node and divide the training set into subsets having the same value for a feature. This process is repeated on all subsets until all the leaf nodes are found. In order to avoid an overfitting [56,57], a process called pruning [58] can be performed at the end of the DT algorithm. The pruning consists of removing leaves and irrelevant branches from the tree [58,59]. In the case of a binary classification of traffic flows in an SDWSN-based IoT, once the most important feature is found using a feature selection technique (e.g., information gain, Gini index), that feature is placed at the root node and used to determine if the traffic flow under consideration is normal or need further considerations to be classified as normal or abnormal. If the traffic flow needs further considerations, the next most important feature is used, and this process is repeated until the traffic flow has been classified as normal or abnormal traffic. The DT algorithm is described below (Box 1).

Box 1 ***Decision Tree Algorithm*.**

1. Allot the most important feature to the root of the tree
2. Divide the training set into subsets having the same value for a feature
3. Repeat the steps above until all the leaf nodes are found
4. End the algorithm when all the leaf nodes are found.

#### 3.3.2. Naïve Bayes

The NB classifier is a predictor that uses the Bayes rule and the Maximum a Posteriori (MAP) rule to predict the label *y* given a feature *X* of a training sample [47,60,61,62]. If the probability is denoted by *P* and the predicted label by $\hat{y}$, then the MAP is given by the equation:

(1) $$\hat{y}=argmaxP\left[y|X\right],$$

Similarly, the Bayes rule is given by the equation:

(2) $$P\left[y|X\right]=\frac{P\left[X|y\right]P\left[y\right]}{P\left[X\right]},$$

The previous equation can be transformed in the equation below:

(3) P[y|X]=P[X|y]P[y],

By substituting Equation (3) into Equation (1), the following equation is obtained:

(4) $$\hat{y}=argmaxP\left[X|y\right]P\left[y\right],$$

For a sample *U* with m features *X*_1_, *X*_2_, *X*_3_, …, *X*_m_, the NB classifier assumes that all features are conditionally independent given the label y. In this case, the previous equation can be rewritten as the equation:

(5) $$\hat{y}=argmaxP\left[U|y\right]P\left[y\right],$$

By substituting the features *X*_1_, *X*_2_, *X*_3_, …, *X*_m_ of the sample *U*, into Equation (5), the following equation is obtained:

(6) $$\hat{y}=argmaxP\left[X_{1},X_{2},X_{3},\ldots ,X_{m}│y\right]P\left[y\right],$$

If two features *X*_1_ and *X*_2_ are conditionally independent given the label *y*, then the following equation can be written:

(7) $$P\left[X_{1},X_{2}|y\right]=P\left[X_{1}|y\right]P\left[X_{2}|y\right],$$

More generally, for m features *X*_1_, *X*_2_, …, *X*_m_, the previous equation can be transformed into the equation below:

(8) $$P\left[X_{1},X_{2},\ldots \;X_{m}|y\right]=\underset{i}{\prod }P\left[X_{i}|y\right],$$

By substituting Equation (8) into Equation (6), the following equation is obtained:

(9) $$\hat{y}=argmaxP\left[y\right]\underset{i}{\prod }P\left[X_{i}|y\right],$$

The prior probability P[y] and the conditional probabilities $P\left[X_{i}|y\right]$ are estimated directly from the training dataset. If the conditional probabilities $P\left[X_{i}|y\right]$ are assumed to be normal distributions, the predictor is called a Gaussian Naïve Bayes classifier [61,63]. Once these probabilities have been estimated, Equation (9) is used as the decision rule to predict the label *y*.

#### 3.3.3. Deep Artificial Neural Network

The connection and behavior of neurons in the brain was responsible for the development of ANNs which try to imitate them [47,60]. In the brain, each neuron receives signals through the synapse. In neuroscience, the connection and the signals send between neurons constitute a biological neural network that influences the global functioning of the brain. The mathematical model of connection in the brain is not well understood yet and for this reason ANNs try to replicate the biological neuron by:

1. Using weights $w_{i}$ on every input value $x_{i}$ to a neuron. Where *i = 1…m*, and *m* is the number of input values;
2. Computing the weighted sum of the input values to the neuron $\sum_{i}^{m}w_{i}x_{i}$;
3. Adding a bias term $w_{0}{}^{i}$ to $\sum_{i}^{m}w_{i}x_{i}$;
4. Using an activation function g to introduce a non-linearity between the input values and the output value of the neuron.

**Figure 3:**
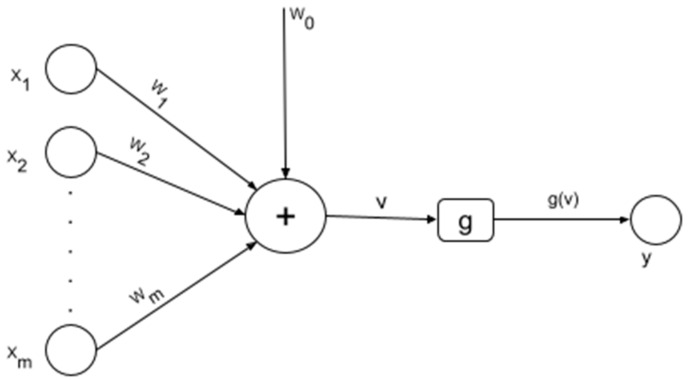
Artificial neuron.

Figure 3 shows the model of an artificial neuron.

**Figure 4:**
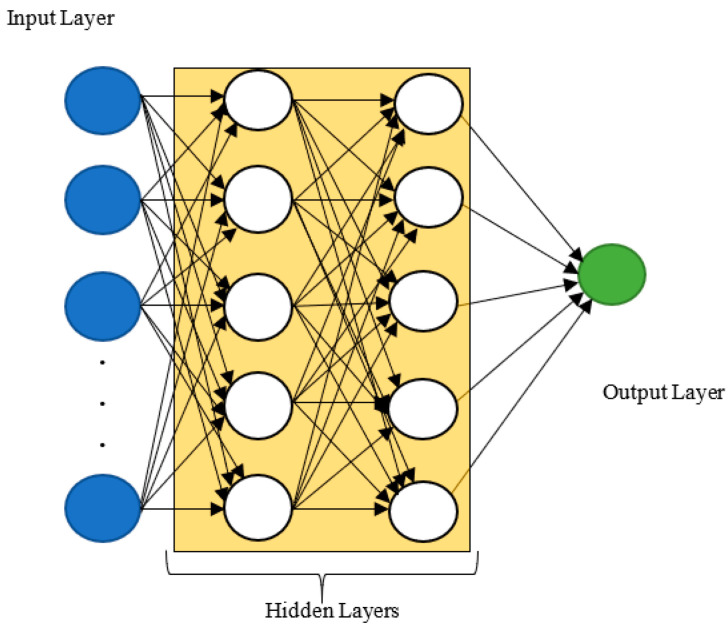
Deep Artificial Neural Network.

The presence of the activation function in the model of the artificial neuron is justified by the need to introduce a non-linearity in the model in order to learn both non-linear and linear functions [64,65]. The most popular activation functions are the sigmoid, the softmax, the tanh, the Rectified Linear Unit (ReLU), the leaky ReLU and the Exponential Linear Unit (ELU) [66,67,68,69,70]. In order to learn very complex functions, artificial neurons can be stack together in layers such that they result in an ANN. An ANN is composed of at least three layers, namely, the input layer, the hidden layer, and the output layer. A deep ANN is an ANN that contains more than one hidden layer. It has been proven that if two ANNs have the same number of neurons but one ANN is deeper than the other, the deepest ANN will tend to yield better performance metrics (e.g., accuracy) unless the vanishing gradient problem occurs (i.e., too deep neural network) [60,71]. Additionally, deep ANNs have the ability to learn any complex function or problem when the size and the hyperparameters are chosen accordingly [60,72]. These facts explain the popularity of deep learning algorithms in general and deep ANNs in particular [73]. Figure 4 shows an example of a deep ANN. 

In order to learn the weights and the biases of an ANN, a cost function $J\left(W_{i},W_{0}{}^{i},{\hat{\;y}}^{\left(i\right)}\right)$ is used to measure how well the predicted outputs ${\hat{y}}^{\left(i\right)}$ are similar to the real values or labels $y^{\left(i\right)}$ of the training dataset. The cost function is also referred to as the loss function or the objective. In the present paper, the cross-entropy loss function will be used given that the goal is the classification of traffic flow in SDWSNs. The cross-entropy loss is given by the equation:

(10) $$J\left(W_{i},W_{0}{}^{i},{\hat{y}}^{\left(i\right)}\right)=-\frac{1}{m}\sum_{i=1}^{m}\left[\left(y^{\left(i\right)}\;\log{\hat{y}}^{\left(i\right)}\right)+\left(1-y^{\left(i\right)}\right)\log\left(1-{\hat{y}}^{\left(i\right)}\right)\right]$$

The weight terms $W_{i}$ and the bias terms $W_{0}{}^{i}$ can be combined in such a way that they form the same weight terms $\theta_{i}$. In this case, the cross-entropy loss is given by the equation:

(11) $$J\left(\theta_{i},{\hat{y}}^{\left(i\right)}\right)=-\frac{1}{m}\sum_{i=1}^{m}\left[\left(y^{\left(i\right)}\log{\hat{y}}^{\left(i\right)}\right)+\left(1-y^{\left(i\right)}\right)\log\left(1-{\hat{y}}^{\left(i\right)}\right)\right]$$

where $y^{\left(i\right)}$ and ${\hat{y}}^{\left(i\right)}$ are, respectively, the actual label of the training example i. m is the number of training examples.

In order to minimize the cost function, the weights $\theta_{i}$ are updated during the training using the gradient descent algorithm [74,75] given by the equation:

(12) $$\theta_{i}=\theta_{i}-\eta \frac{\partial J\left(\theta_{i},{\hat{y}}^{\left(i\right)}\right)}{\partial \theta_{i}}$$

where η is a hyperparameter that need to be tuned adequately to improve the performances metrics.

The gradient of the loss function with respect to each weight is obtained by computing first the gradient of the loss function with respect to the output layer’ s weights and then applying the chain rule to iterate backward up to the first layer’ weights. This process is referred to as backpropagation [60,76,77] in the literature. The vanilla learning process in an ANN is summarized by the following algorithm (Box 2).

Box 2 ***ANN Algorithm*.**

1. Initialize weights $\theta_{i}$
2. Calculate the cost function on the training samples
3. Update the weights $\theta_{i}$ using the gradient descent approach
4. Repeat the steps 2 and 3 until the chosen traditional performance metric does not improve anymore

## 4. Experimental Setup and Results

In order to be able to train the anomaly detectors for multiclass (multinomial) classification, the dataset was preprocessed to contain five labels, namely, normal, U2R, R2L, DoS and Probe. It is noteworthy that the goal of a binary classification is to categorize traffic flows in SDWSNs into two sets, namely, normal traffic flows and attacks. To this end, the U2R, R2L, DoS and Probe labels were replaced by the label “attack” in the dataset in order to be able to train the anomaly detectors for binary classification. More importantly, in order to expect the best performance metrics, the data preprocessing (i.e., normalization, one-hot encoding, feature embedding) was performed and 118 features were derived from the 41 features of the dataset. The 118 features obtained from this end-to-end featuring engineering approach [29,36,78,79,80,81,82] were used to train all of the anomaly detectors developed in this paper.

### 4.1. Binary Classification

#### 4.1.1. NB-Based Anomaly Detector

The default parameters of the Gaussian NB classifier provided in the sklearn library [83,84] were used to train the NB-based anomaly detector. Table 3 gives the metrics recorded while training and evaluating the NB-based anomaly detector.

#### 4.1.2. DT-Based Anomaly Detector

We proceeded analogously to the NB-based anomaly detector’s case by using the default parameters of the DT classifier provided in the sklearn library to train the DT-based anomaly detector. Table 3 gives the metrics recorded while training and evaluating the DT-based anomaly detector.

#### 4.1.3. Deep ANN-Based Anomaly Detector

**Figure 5:**
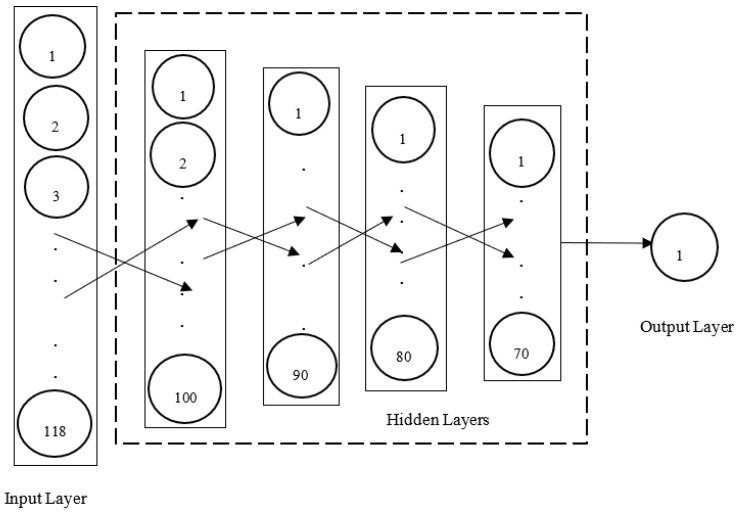
Deep ANN classifier (binary classification).

We used a library called keras [85] to build a deep ANN composed of an input layer with 118 features, four hidden layers with 100, 90, 80 and 70 neurons, respectively, and an output layer with one neuron. The activation functions used for the neurons in the hidden layers were all the ReLU while the activation function used for the neuron in the output layer was the sigmoid function. Given that the output neuron had a sigmoid activation function, it should be noted that the output layer yielded decimal numbers between 0 and 1 which are the probabilities of the output to be a normal traffic. In order to classify an input as normal (i.e., first class), its probability had to be superior to 0.5 and conversely an abnormal input (i.e., second class) had a probability equal or inferior to 0.5 [61,62,66,86]. A grid search approach was used to select the optimal initial learning rate (i.e., 0.00001) by choosing the one leading to the highest performance metrics. Figure 5 shows the deep ANN that was built for the binary classification of traffic flows in SDWSNs. Table 4 indicates the performance metrics achieved by the deep ANN-based anomaly detector for three initial learning rates.

**Figure 6:**
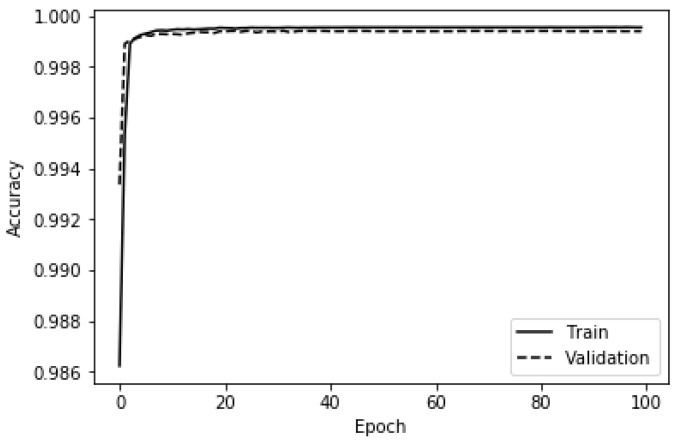
Training and validation accuracy of the deep ANN.

**Figure 7:**
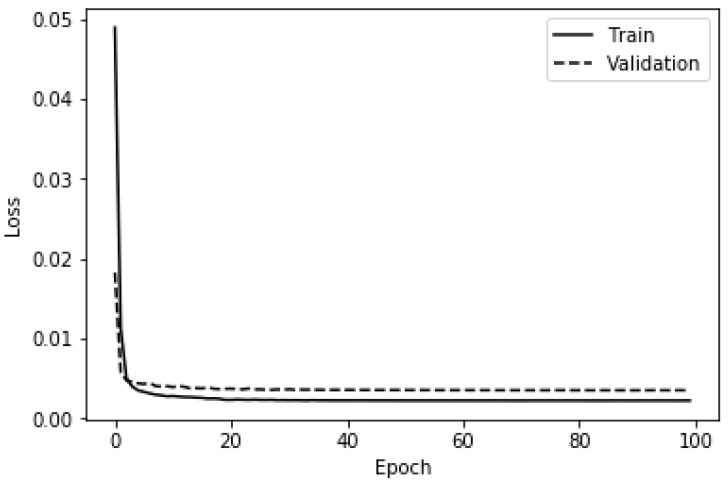
Training and validation loss of the deep ANN (binary classification).

It should be pointed out that in accordance with the best practices in machine learning, a validation set was built by putting aside a quarter of the training set in order to select the optimal hyperparameters (e.g., learning rate reduction factor, initial learning rate) and avoid overfitting [73,87]. In the same vein, the validation loss was monitored, and the learning rate was reduced by a factor of 0.35 if the validation loss did not improve after five successive epochs. Moreover, the maximum number of epochs was fixed to 150 while the minimum learning rate was fixed to 0.0000001. An early stopping was set to occur when the validation loss plateaued after 10 successive epochs. The binary cross-entropy was used as the loss function to keep track of how well the deep ANN was performing. Figure 6 and Figure 7 depict, respectively, the accuracy and the loss yielded by the deep ANN on the train set and the validation set using the aforementioned hyperparameters. It can be concluded from Figure 6 that the overfitting did not occur because the training accuracy and the validation accuracy are almost equal throughout the training phase of the deep ANN. Table 5 gives the metrics recorded while training and evaluating the deep ANN-based anomaly detector.

### 4.2. Multinomial Classification

#### 4.2.1. NB-Based Anomaly Detector

We proceeded analogously to the binary classification case by using the default parameters of NB classifier provided in the sklearn library to train the NB-based anomaly detector. The only difference was that instead of being trained to recognize two classes (i.e., binary classification), the classifier was trained to recognize five classes (i.e., multinomial classification). Table 6 and Table 7 give the metrics recorded while training and evaluating the NB-based anomaly detector for multinomial classification. 

#### 4.2.2. DT-Based Anomaly Detector

Given that the DT-based anomaly detector should be able to perform a multinomial classification, it was trained (using the default parameters in the sklearn library) to recognize the five classes of the training dataset. Table 8 and Table 9 show the metrics recorded while training and evaluating the DT-based anomaly detector for multinomial classification.

#### 4.2.3. Deep ANN-Based Anomaly Detector

**Figure 8:**
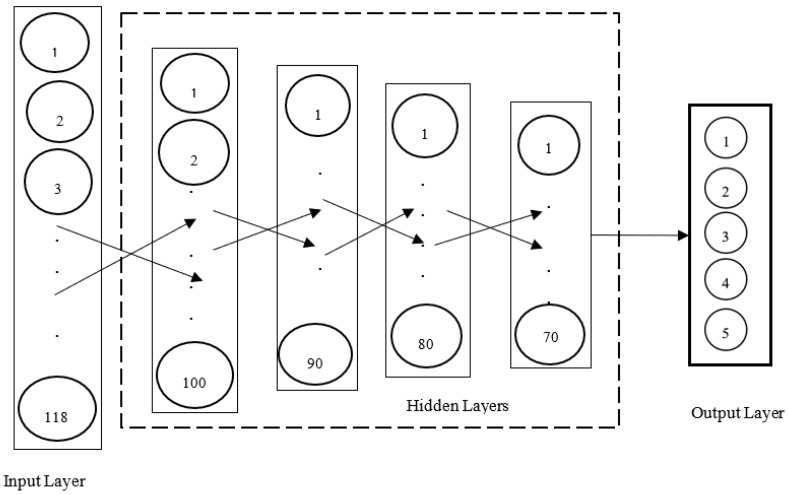
Deep ANN classifier (multinomial classification).

The keras library was once again used to build a deep ANN. The deep ANN was composed of an input layer with 118 features, four hidden layers with 100, 90, 80 and 70 neurons, respectively, and an output layer with five neurons. The activation functions used for the neurons in the hidden layers were all the ReLU while the activation function used for the neurons in the output layer was the softmax. Given that the output layer had five neurons, the class of the input was equivalent to the class of the output neuron that yielded the highest probability [62,86]. Figure 8 shows the deep ANN that was built for the multinomial classification (five classes) of traffic flows in SDWSNs.

**Figure 9:**
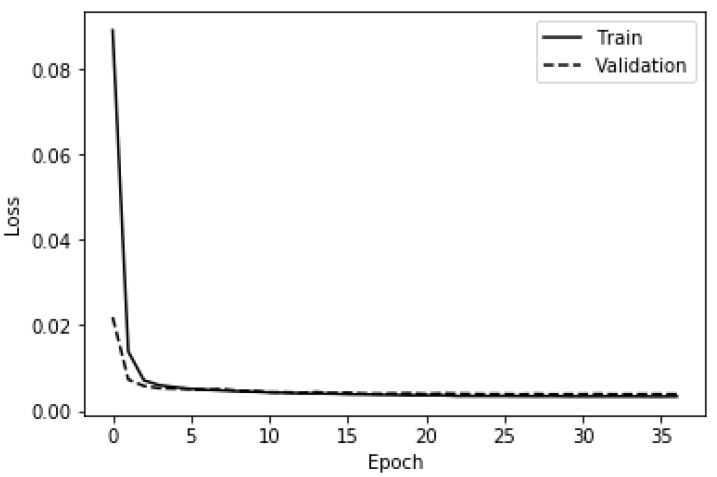
Training and validation loss of the deep ANN (multinomial classification).

The same hyperparameters (i.e., initial learning rate) as in the binary classification case were used to train the deep ANN. The training process was very similar to the binary classification case with the subtle difference that instead of being trained to recognize two classes, the deep ANN was trained to recognize five classes (i.e., multinomial classification). To this end, as previously mentioned, the softmax activation function was used on the five neurons of the output layer. Figure 9 shows the loss of the deep ANN on the train set and the validation set.

From Figure 9, It can be concluded that the hyperparameters used for the training of the deep ANN were adequate because the training loss and the validation loss curves have the same general trend. Table 10 and Table 11 give, respectively, the traditional and nontraditional metrics recorded while training and evaluating the deep ANN-based anomaly detector.

## 5. Summary and Discussion

**Figure 10:**
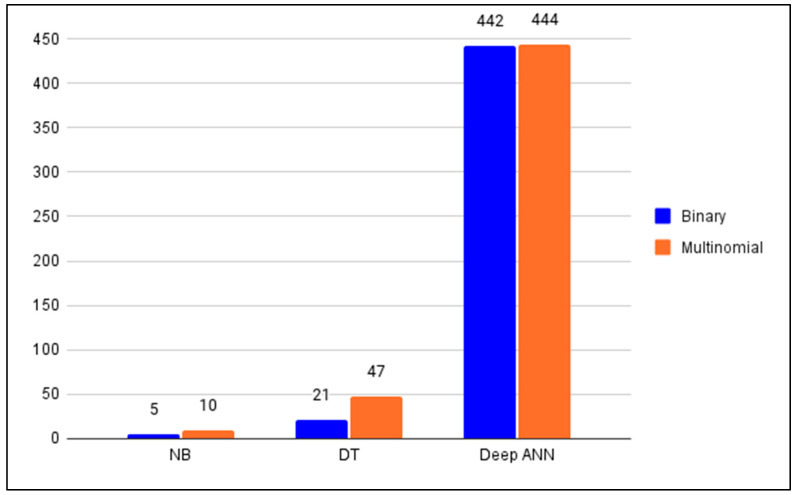
Anomaly detectors’ memory size (kB).

**Figure 11:**
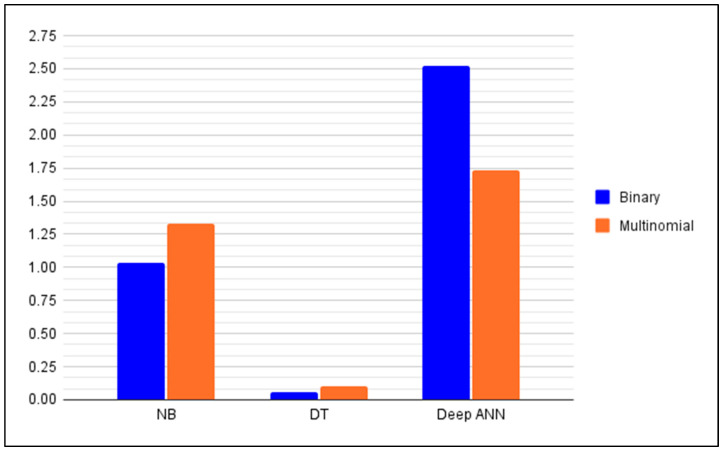
Anomaly detectors’ prediction time (in seconds).

In order to proceed to the discussion, the major results gathered in the previous section are reorganized and summarized in this section into Figure 10 and Figure 11, and Table 12. Figure 10 gives visually the summary of the memory sizes of the anomaly detector models in both the binary classification and the multinomial classification cases. Figure 11 gives the prediction time of the anomaly detector models in both the binary classification and the multinomial classification cases. Table 12 summarizes the metrics recorded during the training of the anomaly detectors in the binary classification case (cf. Table 3 and Table 5).

In the case of the binary classification; by taking into consideration Table 12, Figure 10 and Figure 11; it can be inferred that the NB-based anomaly detector must be preferred in SDWSNs where the memory size of the controller is limited (e.g., small scale or low-power SDWSNs in an African hospital) [3,88]. It should be emphasized that since the higher is the memory size of an anomaly detector the more the controller is energy-intensive, then the NB-based anomaly detector will be the best anomaly detector when the energy consumption is the main concern or the main performance to observe in the SDWSN under consideration [11,13,16,89]. Conversely, if the memory size of the controller is not a concern, the choice of the anomaly detector will be decided between a DT-based anomaly detector and a deep ANN-based anomaly detector. It is noteworthy that, from all three anomaly detectors considered in this paper, the DT-based anomaly detector has the lowest prediction time. For this reason, the DT-based anomaly detector would be preferred in SDWSNs requiring a low latency (e.g., continuous heart monitoring, fall detection in older adults) [3,90,91,92]. Table 13 summarizes the aforementioned considerations. It is noteworthy that the deep ANN-based anomaly detector achieved the same accuracy (i.e., 0.999433) for the binary classification as the LSSVM − IDS + FMIFS framework which was the state-of-the-art IDS found in the literature. More importantly, the DT-based anomaly detector pushed the state-of-the-art accuracy to 0.999777 for the binary classification.

**Figure 12:**
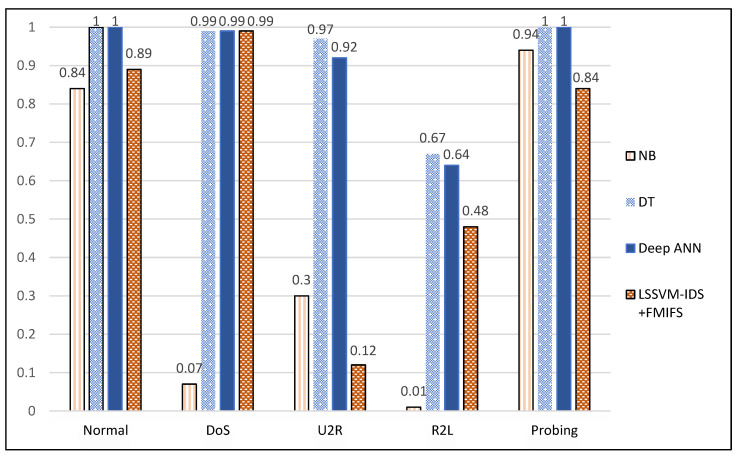
Anomaly detectors’ F-score.

It is noteworthy that the NSL-KDD dataset is inherently imbalanced (e.g., 45927 DoS samples, 52 U2R samples and 995 R2L samples in the training set) and for this reason the most adapted traditional performance metric to evaluate each anomaly detector’ s capability for the multinomial classification is the F-score [93,94]. Similarly to the binary classification case, the memory size and the prediction time will also be considered when making the choice of the anomaly detector the best adapted for an SDWSN under consideration. Figure 12 gives the F-scores (for each of the five classes) of the three anomaly detector models developed in the present paper as well the LSSVM − IDS + FMIFS framework’ s ones. From this figure, it can be seen that the DT-based anomaly detector set new the state-of-the-art F-scores.

In the case of the multinomial classification; by taking into consideration Figure 10, Figure 11 and Figure 12; it can be concluded that the number of training samples play a crucial role in the performance of a classifier. The most striking example is the NB-based anomaly detector that has F-scores of 0.07, 0.3 and 0.01 for the DoS, U2R and R2L attacks, respectively. This means that this anomaly detector cannot be relied upon for the detection of these three attacks in SDWSN-based IoTs even though it can be trusted for the classification of the probing attacks and normal traffics (F-scores of 0.84 and 0.94, respectively). Furthermore, it can be concluded that the DT-based anomaly detector presents the highest F-scores, a reasonable memory size and the lowest prediction time whereas the deep ANN-based anomaly detector presents the biggest memory size. For these reasons, the DT-based anomaly detector should be the default choice when dealing with multinomial anomaly classifications in SDWSN-based IoTs. Additionally, given that the performances of deep learning algorithms in general and deep ANNs in particular increase with the size of the training set, it should be noted that the deep ANN-based anomaly detector would outperform the DT-based one if more U2R and R2L attacks samples could be added to the training set [87,95,96,97]. Finally, given that the miniaturization of the controllers, the ever-increasing memory size of the miniaturized controllers and the fact that deep ANN-based anomaly detector can outperform the DT-based one if more U2R and R2L attacks samples could be added to the training set, the deep ANN classifier should be expected to become in the near future the default anomaly detector in SDWSNs. Table 14 summarizes the considerations drawn from the multinomial classification case. Table 15 gives some examples of IoT applications in healthcare. Table 15 may be used in combination with Table 13 or Table 14 to guide the choice of an adequate anomaly detector.

## 6. Conclusions

In this paper, the NSL-KDD dataset was used to train three classifiers for intrusion detection in IoTs in general and SDWSN-based IoTs in particular. New state-of-the-art accuracy and F-scores have been established by a DT classifier trained on 118 features derived empirically from the 41 features of the NSL-KDD dataset. It was also found that in the case of the binary classification, aside from the memory size, the DT-based anomaly detector presented the best performance metrics and for this reason it should be used as the default anomaly detector in SDWSNs. In the case of small scale or low-power SDWSNs where the memory size of the controller is intrinsically required to be low, the NB-based anomaly detector should be used instead of the DT-based one but with the strong caveat of less security. For this reason, the memory size of the controller should be chosen accordingly when designing SDWSN-based IoTs to avoid compromising data in sensible environments and healthcare application scenarios. In the case of the multinomial classification, it was also found that DT-based anomaly detector presented the best performance metrics and for this reason it should be used as the default anomaly detector in SDWSNs. Additionally, it was found that the NB-based anomaly detector could not be used given its bad performance metrics for the multinomial classification. Finally, given the performance metrics of the deep ANN-based anomaly detector, the memory sizes of this last for both the binomial and the multinomial classification, the ever-increasing number of data collected, the miniaturization of the controllers and the amazing fact the bigger the dataset size, the better the performance metrics of a deep ANN classifier; this last should be expected to become the next default anomaly detector in SDWSNs.

## Figures and Tables

**Table 1:** Traditional performance metrics.

Metric	Symbol	Formula
Accuracy	Ac	$\;Ac=\frac{TP+TN}{TP+TN+FP+FN}$
Precision	P	$P=\frac{TP}{TP+FP}$
Recall	R	$R=\frac{TP}{TP+FN}$
F-score	F	$F=\frac{2}{\frac{1}{P}+\frac{1}{R}}$

**Table 2:** NSL-KDD dataset.

Traffics		Training	Test
**Normal**		67,343	9711
**Attacks**	**DoS**	45,927	7458
	**U2R**	52	67
	**R2L**	995	2887
	**Probing**	11,656	2421

**Table 3:** NB-based and DT-based anomaly detectors’ metrics recorded (binary classification).

Metric	NB-Based	DT-Based
Accuracy	0.948038	0.999777
Precision	0.999114	0.999285
Recall	0.792679	0.999591
F-score	0.884005	0.999438
Prediction time	1.034252 s	0.059382 s
Run time	32.054979 s	26.814881 s
Memory size	5 kB	21 kB

**Table 4:** Performance metrics for different learning rates (binary classification).

Learning Rate	Accuracy	Precision	Recall	F-Score
0.1	0.196240	1.000000	0.196240	0.328095
0.001	0.999021	0.998177	0.996840	0.997508
0.00001	0.999433	0.998830	0.997973	0.998401

**Table 5:** Deep ANN-based anomaly detector’s metrics recorded (binary classification).

Metric	Value
Accuracy	0.999433
Precision	0.998830
Recall	0.997973
F-score	0.998401
Prediction time	2.520133 s
Run time	2 h 20 min 23.361987 s
Memory size	442 kB

**Table 6:** NB-based anomaly detector’s traditional performance metrics recorded (multinomial classification).

Class	Precision	Recall	F-Score
Normal	1.00	0.72	0.84
DoS	0.04	0.94	0.07
U2R	0.23	0.43	0.30
R2L	0.01	1.00	0.01
Probing	0.97	0.91	0.94

**Table 7:** Other metrics recorded (multinomial classification) for the NB-based anomaly detector.

Metric	Value
Prediction time	1.334464 s
Run time	15.390072 s
Memory size	10 kB

**Table 8:** DT-based anomaly detector’s traditional performance metrics recorded (multinomial classification).

Class	Precision	Recall	F-Score
Normal	1.00	1.00	1.00
DoS	0.99	0.99	0.99
U2R	0.96	0.98	0.97
R2L	0.67	0.67	0.67
Probing	1.00	1.00	1.00

**Table 9:** Other metrics recorded (multiclass classification) for the DT-based anomaly detector.

Metric	Value
Prediction time	0.106718 s
Run time	19.176359 s
Memory size	47 kB

**Table 10:** Deep ANN-based anomaly detector’s traditional performance metrics recorded (multinomial classification).

Class	Precision	Recall	F-Score
Normal	1.00	1.00	1.00
DoS	0.99	0.98	0.99
U2R	0.94	0.90	0.92
R2L	1.00	0.47	0.64
Probing	1.00	1.00	1.00

**Table 11:** Other metrics recorded (multinomial classification) for the deep ANN-based anomaly detector.

Metric	Value
Prediction time	1.729457 s
Run time	53 min 23.449426 s
Memory size	444 kB

**Table 12:** Metrics for different anomaly detectors (binary classification).

Metric	NB	DT	Deep ANN
Accuracy	0.948038	0.999777	0.999433
Precision	0.999114	0.999285	0.998830
Recall	0.792679	0.999591	0.997973
F-score	0.884005	0.999438	0.998401
Prediction time	1.034252 s	0.059382 s	2.520133 s
Run time	32.054979 s	26.814881 s	2 h 20 min 23.361987 s
Memory size	5 kB	21 kB	442 kB

**Table 13:** Choice of Anomaly Detectors for SDWSNs (binary classification).

SDWSN Requirements	NB	DT	Deep ANN
High level of security required	NO	YES	YES
Low memory capacity	YES	YES	NO
High performance required (i.e., low latency)	YES	YES	YES

**Table 14:** Choice of Anomaly Detectors for SDWSNs (multinomial classification).

SDWSN Requirements	NB	DT	Deep ANN
High level of security required	NO	YES	YES
Low memory capacity	NO	YES	NO
High performance required (i.e., low latency)	NO	YES	YES

**Table 15:** Requirements and Application Examples.

High Level of Security Required	Low Memory Capacity	High Performance Required (i.e., Low Latency)
▪ Healthcare Data Centers; ▪ Brain Implants; ▪ Medication management through smart pill dispensers; ▪ Smart pulse oximeter; ▪ Alzheimer’s patient tracking and location.	▪ Wearable fitness tracker; ▪ Sleep monitoring system; ▪ Smart infrared body thermometer; ▪ Smart skin moisture analyzer; ▪ Food temperature monitoring system.	▪ Real-time heart monitoring system; ▪ Fall detection system for the elderly people; ▪ IoT-based smart fire alarm system in hospitals; ▪ IoT-based smart light switch and dimmer in healthcare facilities; ▪ Smart infant incubator.

## Data Availability

Not applicable.

## Abbreviations

Abbreviation	Meaning
ANN	Artificial Neural Network
DOS	Denial of Service
DT	Decision Tree
ELU	Exponential Linear Unit
FMIFS	Filter-based Mutual Information Feature Selection
FN	False Negative
FP	False Positive
h	Hours
IDS	Intrusion Detection System
IEEE	Institute of Electrical and Electronics Engineers
IoT	Internet of Things
kB	Kilobytes
LSSVM	Least Square Support Vector Machine
MAP	Maximum a Posteriori
min	Minutes
NB	Naïve Bayes
NSL-KDD	Network Security Laboratory-Knowledge Discovery in Databases
R2L	Remote to Local
ReLU	Rectified Linear Unit
s	Seconds
SDN	Software-Defined Network
SDWSN	Software-Defined Wireless Sensor Network
TN	True Negative
TP	True Positive
U2R	User to Root
WLAN	Wireless Local Area Network
WSN	Wireless Sensor Network
